# Perceived social support and professional identity in nursing students during the COVID-19 pandemic era: the mediating effects of self-efficacy and the moderating role of anxiety

**DOI:** 10.1186/s12909-022-03968-6

**Published:** 2023-02-17

**Authors:** Zhi-Hui Zhao, Jin-Yi Guo, Jie Zhou, Jia Qiao, Shu-Wen Yue, Yan-Qiong Ouyang, Sharon R. Redding, Rong Wang, Zhong-Xiang Cai

**Affiliations:** 1 School of Nursing, Wuhan University, 115 Donghu Rd., Wuchang District, Wuhan, China; 2 Global Health of Project HOPE, Maryland, USA; 3 Renmin Hospital of Wuhan University, Wuhan, China; 4 Nursing Department, East Campus of Renming Hospital of Wuhan University, Gaoxin Sixth Road, Jiangxia District, Wuhan, China

**Keywords:** Self-efficacy, Social support, Mediation, Nursing students, Professional identity

## Abstract

**Background:**

Health professionals, including nurses, experienced heavy workloads and significant physical and mental health challenges during the coronavirus disease (COVID) 19 pandemic, which may affect career choices for those considering nursing and for nursing students. The COVID-19 pandemic is not only a period of risk, but also an occasion to redeploy the professional identity (PI) of nursing students. However, the relationship between perceived social support (PSS), self-efficacy (SE), PI and anxiety remains unclear under the background of COVID-19. This study aims to explore whether PSS has an indirect effect on PI through mediation of SE and whether the anxiety can moderate the relationship between PSS and SE in nursing students during their internship period.

**Methods:**

An observational, national cross-sectional study was conducted following the STROBE guidelines. An online questionnaire was completed by 2,457 nursing students from 24 provinces in China during their internship during September to October 2021. Measures included Chinese translations of the Professional Identity Questionnaire for Nursing Students, the Perceived Social Support Scale, the General Self-Efficacy Scale, the 7-item Generalized Anxiety disorder scale.

**Results:**

Both PSS (*r* = 0.46, *p* < 0.001) and SE (*r* = 0.51, *p* < 0.001) were positively correlated with PI. The indirect effect of PSS on PI through SE was positive (*β* = 0.348, *p* < 0.001), with an effect of 72.7%. The results of the moderating effect analysis showed that anxiety attenuated the effect of PSS on SE. Moderation models indicated that anxiety has a weak negative moderating effect on the effect of PSS on SE (*β* =—0.0308, *p* < 0.05).

**Conclusions:**

A better PSS and higher scores in SE were associated with PI in nursing students, and a better PSS had an indirect effect on the PI of nursing students through SE. Anxiety played a negative moderating role in the relationship between PSS and SE.

**Supplementary Information:**

The online version contains supplementary material available at 10.1186/s12909-022-03968-6.

## Introduction

Nurses play an increasingly important role in the entire health system and are at the heart of the patient care process, acting as a link between patients, families and physicians. However, according to the WHO State of the World’s Nursing 2020 report, the global nursing workforce shortage will be up to 4.6 million by 2030 if no action has been taken [1]. Moreover, during the COVID-19 pandemic, nurses underwent heavy workloads and significant physical and mental health challenge[2]. Changes in the healthcare system and the quest for quality care are placing greater demands on the role of the nurse [3], which these may influence career options for nursing students. As the reserve force of future nursing talents, the career choice of nursing students affects the number of nursing teams to a great extent.

Professional identity (PI) in nursing is a self-concept derived by the individual about the role of the nurse, which is a combination of personal values, attitudes and beliefs and the characteristics and understanding of the nursing profession [3]. PI is a dynamic and flexible process, the progressive development and advancement of PI is a key factor in the provision of health care [4]. And PI is a strong predictor for job retention in nursing profession [5]. Some studies have demonstrated that a deficiency of PI in nursing students probably increases stress, reduces self-confidence and decision-making capacity, and nursing students are more likely to believe others' definitions of their profession [6]. Stetson et al. (2020) noted that COVID-19 induced changes may affect medical students' professional identity formation [7]. Researches indicated that the COVID-19 pandemic had a positive impact on nursing students' PI [2, 8, 9]. Pre-pandemic COVID-19, 50.9% of Chinese nursing students showed that they chose nursing as their intended career, while the corresponding figure increased to 62.7% after the pandemic [2], both of these figures are higher than the results of a previous study in Taiwan (34.6%) [10]. However, they are significantly lower than the equivalent amounts for the United States (99.4%) and Turkey (81.1%) [11]. This may be related to the fact that nurses are portrayed in the media as heroic, self-sacrificing and having a strong sense of morality.

The COVID-19 pandemic is not merely a time of crisis but also an special opportunity to reconstruct the PI of nursing students [12]. The PI of nursing students is influenced by many factors, including clinical learning environment, job positions of parents, psychological condition [9, 13]. When the COVID-19 epidemic was severe for a certain period, Chinese nursing students were temporarily removed from all clinical placements in training hospitals which may have led to nursing students experiencing feelings of inadequacy and thus affecting the PI [12]. Perceived social support refers to an individual’s expectations, feelings and evaluation of social support situations [14]. Social support refers to the support provided by family, friends and others which could include peers, guidance by a teacher, formal and informal education opportunities and instruction in a hospital setting [15]. Research has shown that nursing students' PI is positively correlated with the level of social support they receive from all walks of life [16]. Hence, increasing the sources and breadth of social support for nursing students can have a positive effect on fostering an increase in their PI.

The large number of health care workers infected during the COVID-19 pandemic and the enormous work pressure on clinical nurses caused nursing students to show anxiety and fear about their PI in nursing profession [17]. Anxiety is a negative emotion, which mainly consists of feelings such as unease, worry, doubt and fear [18]. For nursing students who will enter the clinic need to confront situations that put them at high risk, which greatly increases their anxiety levels. Moreover, one of the most important is the anxiety can affect nursing students' attitude towards the nursing profession [19]. During the epidemic, acute and life-threatening pressure caused by infectious disease events changed nursing students’ PI [20]. Sun’s (2020) study of 474 nursing students found that the prevalence of anxiety was 12.4% during the COVID-19 pandemic [21]. Nursing students' attitudes towards their profession and their level of anxiety are relatively unknown in this unprecedented era.

According to the self-efficacy theory [22], the core of what influences people's behaviour is their belief in their ability to behave and people always tend to choose activities in which they have confidence to succeed. SE is considered to be a dynamically constructed process, influenced by new experiences, in the COVID-19 pandemic, nursing students' SE is altered by changes in the educational and clinical environment [23]. Many studies have shown that SE of nursing students is positively correlated with PI [24–28]. The higher the SE, the stronger the learning interest and enthusiasm of nursing students, which can promote their correct understanding of the social value and development prospect of nursing, so as to obtain a higher PI [26, 29].

For nursing students, PI is an important belief as it influences not only students' perceptions of their profession, but also their learning behaviour and even their willingness to enter the nursing profession [30]. Recognizing the factors that affect PI is essential to implementing effective strategies to enhance students' career well-being in such a time of uncertainty [31]. However, no studies have been aimed at mapping the complex relationship between PSS, SE, anxiety and PI, especially during the context of the Covid-19 pandemic. Thus, this study tested a conceptual model to fill the gap whether SE and the anxiety provide informative pathways in the association between PI and PSS. According to the findings of previous studies, the following hypotheses are proposed in this study:

- Hypothesis 1 The variable PSS may be directly associated with PI.
- Hypothesis 2 The PSS is directly correlated with SE.
- Hypothesis 3 The existence of stronger SE among nursing students is associated with higher levels of better PI.
- Hypothesis 4 The SE may have a mediating role between PSS and PI. Higher PSS scores might be indirectly associated with better PI through stronger SE.
- Hypothesis 5 Anxiety may play a moderate role between PSS and SE.

## Methods

### Study design

An observational, national cross-sectional design was used and this study was implemented with the STROBE checklist (see Supplementary material 1).

### Participants

A total of 2,737 respondents were recruited for the online survey platform during September to October 2021. The inclusion criteria were as follows: (a) full-time nursing students completing a practicum internship at a teaching hospital; (b) completing an associate degree, baccalaureate or master’s degree nursing programme; (c) ability to use smartphones or computers. The exclusion criteria was being absent from the internship for more than two weeks due to sick leave or completing a job search.

### Setting and data collection

In this study, with consideration of the representativeness of the participants, 24 provinces in seven geographical regions which they are Northeast China, North China, central China, East China, northwest China, southwest China and South China were chosen using a multi-stratified random sampling method. Faculty members at nursing programs known by the researchers were identified and contacted by the research group. Electronic copies of the informed consent form were sent to participants via email and they were notified of the objectives of the research and the eligibility of the participants.

In this study,“questionnaire star”online platform was applied to complete the online survey (accessed at www.wjx.cn). Participants accessed the survey by clicking on the survey link or scanning the QR code using a smartphone or computer and took approximately 15 min to complete. The inclusion and exclusion criteria were explained in the questionnaire link and QR code. Participants who did not answer all questions, completed the survey in less than 15 min, or had contradictory items in the reverse order were excluded from the data analysis. Participants could only complete one survey based on their Internet-protocol (IP) address.

### The calculation of the sample size

The minimum sample size required was calculated using power analysis and sample size software (PASS, version 11.0) with a two-sided 95% confidence interval and a mean to limit distance of 1.5, SD of 11.3 [9]. The minimum sample size required was 266, with a dropout rate of 20%.

### Measurements

#### Socio-demographic information

A socio-demographic questionnaire developed by the research team was used to collect basic information, family background, school performance, attitude toward the nursing profession and a nursing career and knowledge and attitude about the COVID-19 pandemic (See Table 1).

**Table 1:** Socio-demographic characteristics of participants (*N* = 2,457)

Characteristics	Number	%
Gender		
Male	276	11.2%
Female	2181	88.8%
Only child		
Yes	633	25.8%
No	1824	74.2%
Age(in years)		
< 18	353	14.4%
18–21	1688	68.7%
22–25	404	16.4%
26–30	12	0.5%
Family structure		
Parental family	2059	83.8%
Single parent family	225	9.2%
Reconstituted family	117	4.8%
Other	56	2.2%
Birthplace		
Rural	1813	73.8%
City	644	26.2%
Monthly available disposable living expenses(¥, Yuan)		
< 1000	687	28.0%
1000 ~	1261	51.3%
1500 ~	377	15.3%
2000 ~	76	3.1%
≥ 2500	56	2.3%
Overall economic status of family		
Bad	694	28.2%
Poor	629	25.6%
Average	999	40.7%
Good	110	4.5%
Excellent	25	1.0%
Internship duration (in months)		
0–3	1661	67.6%
4–6	558	22.7%
7–9	238	9.7%
Nursing program		
Associate's degree	1264	51.5%
Bachelor’s degree	1094	44.5%
Master’s degree	99	4.0%
Academic performance		
Good (top third of class)	639	26.0%
Medium (middle third of class)	1742	70.9%
Poor (bottom third of class)	76	3.1%
Whether to hold student officer positions		
Yes	701	28.5%
No	1756	71.5%
Whether nursing is their first choice		
Yes	1902	77.4%
No	555	22.6%
Satisfaction with current nursing work		
Very satisfied	402	16.4%
Moderately satisfied	1067	43.4%
Satisfied	884	36.0%
Dissatisfied	79	3.2%
Very dissatisfied	25	1.0%
Whether parents are health care workers		
Yes	71	2.9%
No	2386	97.1%
Whether there are suspected or confirmed cases of COVID-19 in the place of residence during the pandemic		
Yes	217	8.8%
No	2240	91.2%
Perception of the pandemic		
Very concerned	1687	68.7%
Concerned	754	30.7%
Don't pay much attention	14	0.6%
Pay no attention	2	0.08%
Will you contribute to the fight against COVID-19		
Yes	2361	96.1%
No	96	3.9%
Whether you still plan to consider a nursing career in the future		
Yes	2214	90.1%
No	243	9.9%

#### The Professional Identity Questionnaire for Nursing Students (PIQNS)

The Professional Identity Questionnaire for Nursing Students (PIQNS) developed by Hao Yufang (2011) was used in this study. The questionnaire included 17 items in five dimensions: career self-concept (items 1, 6, 9, 11, 16, 17), retention benefits and exit risks (items 5, 8, 10, 14), social comparison and self-reflection (items 7, 13, 15), career choice and autonomy (items 4, 12) and social persuasion (items 2, 3). 16 items were scored on a five-point Likert scale (strongly disagree, disagree, uncertain, agree, strongly agree) with item 12 being reverse scored and a total score of 85. The greater the score, the more positive the professional identity. The scale has good reliability and validity with a Cronbach's alpha of 0.827 and a split-half reliability of 0.842. The scale has been widely used in measuring the professional identity of nursing students in China [32].

#### Perceived Social Support Scale (PSSS)

The Perceived Social Support Scale (PSSS), designed by Zimet, Dahlem, and Farley in 1988, was used to assess participants' perceived social support [33] and had been translated into Chinese by Li et al. [15, 34]. It is composed of 12 items with response choices from one point (very strongly disagree) to seven point (very strongly agree). The PSSS assesses the quality of social support in three areas: family, friends and significant others. Scores for all items are totaled and divided by 12. The mean scores range from 1–2.99, 3–5 and 5.01–7 indicating low, medium and high levels of perceived support, respectively. The Chinese version of the PSSS showed good reliability and validity in different Chinese populations, with Cronbach's alpha of 0.92 [15].

#### General Self-Efficacy Scale (GSES)

The General Self-Efficacy Scale (GSES) was developed by Schwarzer et al. in 1995 and translated by Caikang Wang et. al. in 2001 [35]. The scale covers some of the psychological states and behaviors that individuals may occur when facing difficulties or setbacks, etc. [36]. Ten of these items were ranked on a four-point Likert scale ranging from "strongly disagree" to "strongly agree". There are three levels of self-efficacy: low (10–20 points), medium (21–30 points) and high (31–40 points). Higher total score means better self-efficacy. For the Chinese version of the GSES, the Cronbach’s alpha reliability coefficient was 0.87. A split-half reliability was 0.78 and the retest reliability was 0.83. The correlation coefficients of the 10 items and the total scale in terms of validity were 0.60–0.70. The scale showed good reliability and validity and is widely used in China [37].

#### The 7-item Generalized Anxiety disorder scale (GAD-7)

The GAD-7 was a brief anxiety symptom self-rating scale developed in 2006 by Spitzer et al. based on the Diagnostic and Statistical Manual of Mental Disorders Fourth Edition (DSM-IV) symptom criteria to assess the frequency of anxiety symptoms [38]. The scale consists of 7 items on a four-point Likert scale as follows: not at all (0), a few days (1), more than half of the days (2) and almost every day (3). The total score range is 0–21 and the scores 5,10,15 are the cut-off values for mild, moderate and severe anxiety levels, respectively. It was translated into Chinese by Li et al. in 2010 and its Cronbach's α coefficient of the scale was 0.898 [37]. Chinese version of the GAD-7 showed good reliability and validity and is widely used in China for assessing anxiety level [2].

### Data analysis

SPSS version 22.0 was used for the data analysis performed and use Hayes’ PROCESS macro (Model 4) of SPSS to analyze the mediation analysis. The moderated mediating effect of nursing students' anxiety on PSS and SE was examined using Hayes’ PROCESS macro for SPSS (Model 7). Pearson’s correlation analysis between PSS, SE, PI and anxiety were analyzed to determine if mediation and moderation analysis were required. Additionally, bootstrap methods were used to test the significance of all effects to obtain robust standard errors for the parameter estimates [39]. The bootstrap method produced 95% bias-corrected confidence intervals for these effects derived from a resample of 5,000 data. A confidence interval that does not include zero indicates that the effect is significant [40].

## Results

A total of 2,737 nursing students completed the survey, however, 280 invalid questionnaires were removed due to either (a) too short a response time or (b) contradictions in the questionnaire's reverse questions. The final available data for analysis was 2,457, with a response rate of 89.8%. Their average age was 19.3 years old ranging from 18 to 26 and 88.8% were female. The only child ratio was 25.8%. Of the participants, 26.2% resided in cities and 73.8% in rural areas. The average scores of the GSES, PSSS, PIQNS and GAD-7 were 26.53 ± 6.93, 61.67 ± 13.55, 64.00 ± 12.78 and 3.74 ± 4.62 respectively (see Table 2).

**Table 2:** Descriptive statistics and correlations of four variables (*N* = 2,457)

Variables	M	SD	Perceived social support	Self-efficacy	Professional identity	Anxiety
Perceived social support	61.67	13.55	1			
Self-efficacy	26.53	6.93	0.55**	1		
Professional identity	64.00	12.78	0.46**	0.51**	1	
Anxiety	3.74	4.62	-0.29**	-0.17**	-1.90**	1

*Abbreviations*: *M* mean, *SD* standard deviation

^**^p < .001

All *p* values are 2-tailed

### Correlation analyses

Table 2 demonstrates Pearson's two-tailed correlation coefficient of the PSS, SE, PI and anxiety in the study. There is a significant positive correlation between PSS, SE, and PI. Anxiety is negatively correlated with PSS, SE and PI. Nursing students with high PSS were more likely to have higher levels of SE and stronger PI.

### Test of mediating effect

The direct path coefficients investigated by all models in this study are presented in Table 3. PI was entered as the dependent variable in the multiple linear regression analysis, demographic variables as control variables and PSS and SE as the main predictors. The direct and indirect paths of the PSS and PI, as well as the path size of the modulating effect of the PSS on the PI through the SE, are calculated. The total effect of PSS on PI explained 25.8% of the variance and the total effect was significant (*c* = 0.479, *p* < 0.001) as demonstrated in Table 3.

**Table 3:** Mediation model and effects of the PSS and SE on PI (*N* = 2,457)

Model	Path		Informant				
			***β***	***SE***	***p***	**95% CI**	**R**^**2**^
Model 1	c (total effect)	Perceived Social Support → Professional Identity	0.479	0.016	< 0.001	0.446 ~ 0.511	0.258
Model 2	a	Perceived Social Support → Professional Identity	0.281	0.008	< 0.001	0.264 ~ 0.298	0.302
Model 3	b	Self-Efficacy → Professional Identity	0.467	0.037	< 0.001	0.393 ~ 0.539	-
	c' (direct effect)	Perceived Social Support → Professional Identity	0.348	0.019	< 0.001	0.310 ~ 0.385	
Bootstrap estimate	Indirect effect	Perceived Social Support → Self-Efficacy → Professional Identity	0.131	0.013	< 0.001	0.105 ~ 0.157	

*Abbreviations*: *SE* Standard error, *CI* confidence interval, Perceived Social Support, *SE* Self-Efficacy, *PI* Professional Identity

**Fig. 1:**
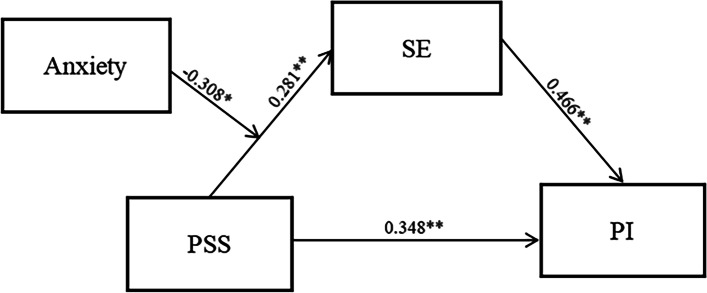
Mediation model. SE, Self-Efficacy; PSS, Perceived Social Support; PI, Professional Identity; **p < .001, *p < 0.05

The SE mediated relationships between PSS and PI were performed through the PROCESS macro of SPSS 22 using Model 4. The path relationships between the variables in this study are shown in Fig. 1. When demographic variables were analyzed as control variables, SE explained 30.2% of the variance in the moderation of PSS and PI and the Model 4 was significant under bootstrapping test (*F* = 531.028, *p* < 0.001, *R*^*2*^ = 0.302). Regression analysis showed that nursing students with greater PSS showed higher SE, while nursing students with higher SE had stronger PI, consistent with the results of Model 4 path analysis.

This study took bootstrap to test the mediating effect of SE between PSS and PI and the results showed that higher PSS was associated with stronger PI through better SE. The results show a significant mediating effect of the indirect effect of PSS acting on PI through SE (*β* = 0.131), as based on 5,000 bootstrap samples with no excess of zero (95% CI 0.105 to 0.157), confidence intervals that do not contain zero indicate a significant effect. Moreover, the direct effect of PSS on PI was verified (*c'* = 0.348, *p* < 0.001) (95% CI 0.105 to 0.157). Finally, the hypothesis 4: the role of SE as a partial mediator between PSS and PI is also fully confirmed.

### Test of mediating moderation effect

**Fig. 2:**
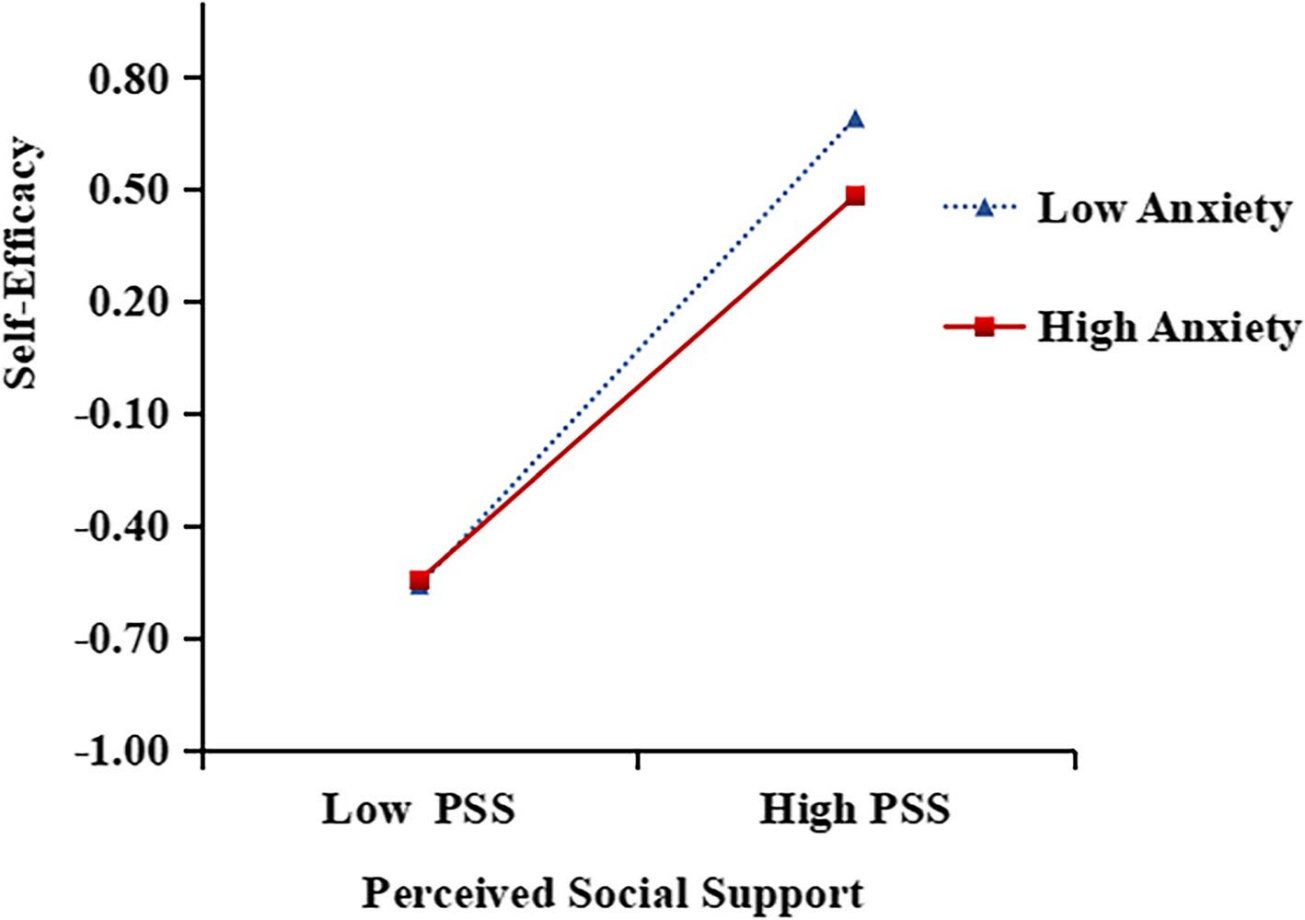
The moderating effect of the anxiety on the relationship between perceived social support and self-efficacy

The moderating effect of anxiety was further analyzed by a simple slope test using Model 7 of the PROCESS [39] macro (see Table 4). The high and low anxiety groups were determined by a moderating variable, which was based on the mean score of anxiety plus or minus one standard deviation (see Fig. 2). When the level of anxiety was low, with the increase in PSS, the level of SE of nursing students increased significantly (*β* = 0.571, *p* < 0.001). When the level of anxiety was high, with the improvement of PSS, the level of SE of nursing students increased significantly (*β* = 0.515, *p* < 0.001). This suggests that the effect of PSS on SE decreases with the increase of anxiety level. Therefore, hypothesis 5 is also confirmed.

**Table 4:** The moderated mediating effect of anxiety on PSS and SE (*N* = 2,457)

Dependent variable model (Outcome variable:Self-efficacy)						
Variables	Estimate	*SE*	*t*	*p*	LLCI	ULCI
Constant	-0.009	0.017	-0.505	0.614	-0.043	0.025
Perceived Social Support	0.545	0.018	31.009	0.000	0.511	0.580
Anxiety	-0.023	0.018	-1.303	0.193	-0.059	0.012
Perceived Social Support × Anxiety	-0.031	0.016	-1.980	0.048	-0.061	-0.001
R^2^ with interaction		*R*^*2*^		*F*		*p*
		0.001		3.920		0.047

*Abbreviations*: *SE* Standard Error, *LLCI* lower limit of 95% confidence interval, *ULCI* upper limit of 95% confidence interval, *SE* Self-Efficacy, *PSS* Perceived Social Support

## Discussion

Although numerous studies have explored factors affecting nursing students' PI [29, 41, 42] few have examined the role of mediators and moderating roles [43]. In the particular case of the COVID-19 pandemic, this study successfully excavated the internal specific mechanism of interaction among the PSS, SE, PI and anxiety of nursing students during their internship. There are four variables considered for the first time in this study when applying this model. This study revealed that the total direct effect of PSS on PI was 72.629%. Self-efficacy explains part of the effect of PSS on PI, and SE acts as an important mediator between PSS and PI. The total indirect effect is 27.372% of the total effect. Importantly, there is a moderating effect of anxiety on the relationship between PSS and SE. Therefore, the findings of the study are presented below: (a) Nursing students who have a higher sense of PI are those who had more PSS; (b) Nursing students with greater PSS show greater SE and a greater sense of PI; (c) PSS positively predicts self-efficacy, which would then positively predict PI; (d) The anxiety of nursing students plays a moderating role in the relationship between PSS and SE.

This study demonstrated that PSS can directly affect PI and positively correlated with the SE (Tables 2 and 3) and further validated the theory of social support of Lin [44]. Based on the theory of social support, Sarason, Yue, and Yong argued that PSS plays a significant role in explaining the mechanism between the positive support system of PSS and PI [45–47], which were aligned with this study regarding the association between PSS and PI. Sabatino's research pointed out that the optimistic and positive supportive atmosphere provided by departmental teachers has an extremely important impact on the professional values of nursing students [48]. Accordingly, healthcare institutions should promote a staff teaching model, provide educational resources for nurses serving as clinical instructors, coordinate education programs in collaboration with nursing faculty at schools of nursing.

The results of this study reflected SE strongly mediates the link between PSS and PI, which indicated that nursing students' sense of SE could improve their PI. This finding was consistent with previous studies of college students [49–53]. Lent et al. developed social cognitive career theory (SCCT) in 1994 [54] which was the specific application of Bandura’s self-efficacy theory in the professional field. According to the SCCT, researcher has found that there is a positive correlation between social support, SE and PI [55]. Mohammad’s study has proved that parental authority also mediated the relationship between career decision-making self-efficacy and influenced nursing career choice [43]. In this study, self-efficacy only provides part of the mediating effect, and there may be additional potential mediating effects that have not yet been identified. This suggests that further research is required to ascertain the underlying factors impacting SE on nursing intern students.

A key finding is that anxiety was identified as a moderator between PSS and SE in this current study. The correlation between PSS and SE was weaker for internship nursing students with anxiety compared to students without anxiety in the light of the ongoing Covid-19 epidemic. According to Bandura's Four Paths, anxiety is the enemy of self-efficacy, and as anxiety levels rise, self-efficacy levels decline [56]. Furthermore, previous studies have shown that students' anxiety during the Covid-19 pandemic had a negative impact on their professional identity in nursing [21, 57, 58]. Given the potential negative effects of COVID-19 on nursing students inducing anxiety and other harmful mental health effects [58]. It has been shown that the flipped approach can work against anxiety-provoking components and increase nursing students' self-efficacy [59]. A systematic review has illustrated the effectiveness of anxiety interventions such as guided reflection, training using the Emotional Freedom Technique and mindfulness programs [58]. Researchers could implement measures to increase nursing students' self-efficacy and counteract the negative effects of anxiety.

### Implications for clinical practice

The current study has found that PSS could not only directly predict PI, but also indirectly predict PI by improving SE as a mediating variable. Therefore, in the clinical internship stage, nurse educators should be aware that focusing on improving PSS may be a path to improve PI, and more importantly, it may also be the path to improve SE. The current research findings have shown that the PSS, SE and PI of nursing students were at a moderate level during the Covid-19 epidemic, which is consistent with some previous studies [49, 52, 55, 60].

The pandemic has been shown to have a positive effect on the PI of nursing students [2, 9]. Understanding this pandemic and integrating relevant findings into educational programs has the promise to promote PI among nursing students and increase their engagement in staying in the nursing profession [12]. As for the intervention program strategies for PSS, nurse educators should support and promote the social network of nursing students through "group learning", "one-to-one teaching", "class psychological construction" and so on, and promote the establishment of mutual trust between teachers and students [61, 62]. According to Bandura's self-efficacy theory, SE is mainly affected by substitute experiences, verbal persuasion, success or failure experiences, emotional states and other factors [22], which suggests that nurse educators should focus on the above four dimensions in teaching activities to promote clinical competence in the new nursing graduate. In the context of the current COVID-19 pandemic, nurse educators could strengthen PI by inviting front-line or senior nurses to serve as role models to promote nursing students’ SE and PI [63, 64].

In conclusion, this study has bridged the gap in the literature on the relationship between PSS, SE, PI and anxiety which also set a theoretical foundation for future researches.

### Strength and limitations

This is the first multi-center and large sample study to determine the mediating role and moderating role in the relationship among PSS, SE, PI and anxiety for nursing students in twenty-four provinces in China, in the broader perspective of the COVID-19 pandemic. This enhances the generalizability of findings to other provinces and possibly other countries in Asia. Several limitations need to be acknowledged. Firstly, this study used a cross-sectional survey, which can’t explain causality. Secondly, self-efficacy only provides part of the mediating effect, and there may be additional potential mediating effects that have not yet been identified. Therefore, longitudinal studies will be needed in the future to explore the true causal relationship and qualitative interviews could be considered to gather other data to explore potential mediating factors.

## Conclusions

This study has revealed that PSS has an indirect effect on PI through mediation of SE and the anxiety can moderate the relationship between PSS and SE in nursing students. Moreover, SE of student may be threatened by anxiety, which in turn adversely affects PI in nursing students. According to these findings, nurse educators, researchers and nurses in administrative roles in practice settings should pay attention to the impact of multi-dimensional perception of social support and SE interventions on PI.

## Supplementary Information

**Additional file 1.**

## Data Availability

Data used and/or analyzed during the current study are available from the corresponding author on reasonable request.

## Abbreviations

PSS: Perceived social support
PI: Professional identity
SE: Self-efficacy
COVID: Coronavirus disease
PIQNS: The Professional Identity Questionnaire for Nursing Students
PSSS: The Perceived Social Support Scale
GSES: The General Self-Efficacy Scale
GAD-7: The 7-item Generalized Anxiety disorder scale
IP: Internet-protocol
